# Supplementary material for: Single-cell lung eQTL dataset of Asian never-smokers highlights the roles of alveolar cells in lung cancer etiology
Source: bioRxiv. 2026 Mar 27:2026.03.26.714500. Preprint. [Version 1] doi: 10.64898/2026.03.26.714500 (PMC13041904; doi:10.64898/2026.03.26.714500)
Supplement: Supplement 2 [file NIHPP2026.03.26.714500v1-supplement-2.pdf]

bioRxiv preprint doi: https://doi.org/10.64898/2026.03.26.714500; this version posted March 27, 2026. The copyright holder for this preprint (which was not certified by peer review) is the author/funder. This article is a US Government work. It is not subject to copyright under 17 USC 105 and is also made available for use under a CC0 license.

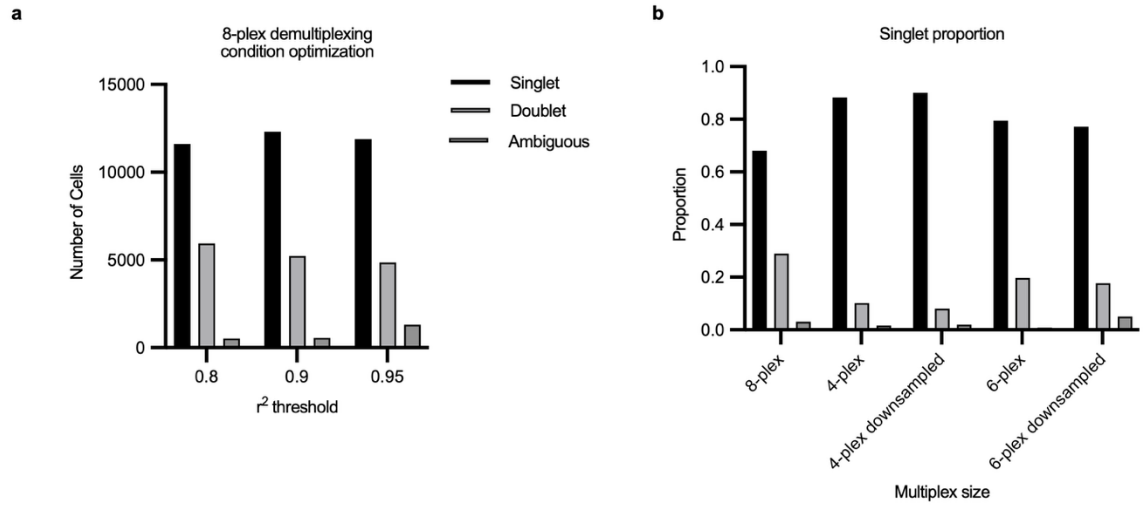

Supplemental Figure 1. Multiplexing optimization.
Sample multiplexing batch size was determined based on the efficiency of the genotype-based demultiplexing to achieve the maximum recovery of cells assigned as singlets and reduce the cost. Germline genotype data from matched blood samples was imputed using Michigan imputation server and 1000 genomes EAS populations as a reference. The imputed genotypes were then filtered by quality (imputation $r^2 > 0.8$ to 0.95) and utilized by Demuxlet to match the genotypes from scRNA-sequencing of each cell to assign samples to cells. **a,** Genotype imputation quality cutoffs were compared between $r^2 > 0.8$, 0.9, and 0.95 with an alpha = 0.5 in an 8-plex setting. The cutoff with the maximum singlet cells ($r^2 = 0.9$) was chosen. Bar ordered follows legend. **b,** 8-plex vs 4-plex were compared for singlet recovery at $r^2 > 0.9$ and alpha = 0.5. To account for sequencing depth differences, we also included a down-sampled 4-plex with a matching read-depth to the 8-plex sample. Considering the singlet proportion and sequencing cost, we chose 6-plex, aiming to achieve ~80% singlet (between 8-plex and 4-plex). A representative 6-plex sample alongside the down-sampled one matching the 8-plex is shown.

bioRxiv preprint doi: https://doi.org/10.64898/2026.03.26.714500; this version posted March 27, 2026. The copyright holder for this preprint (which was not certified by peer review) is the author/funder. This article is a US Government work. It is not subject to copyright under 17 USC 105 and is also made available for use under a CC0 license.

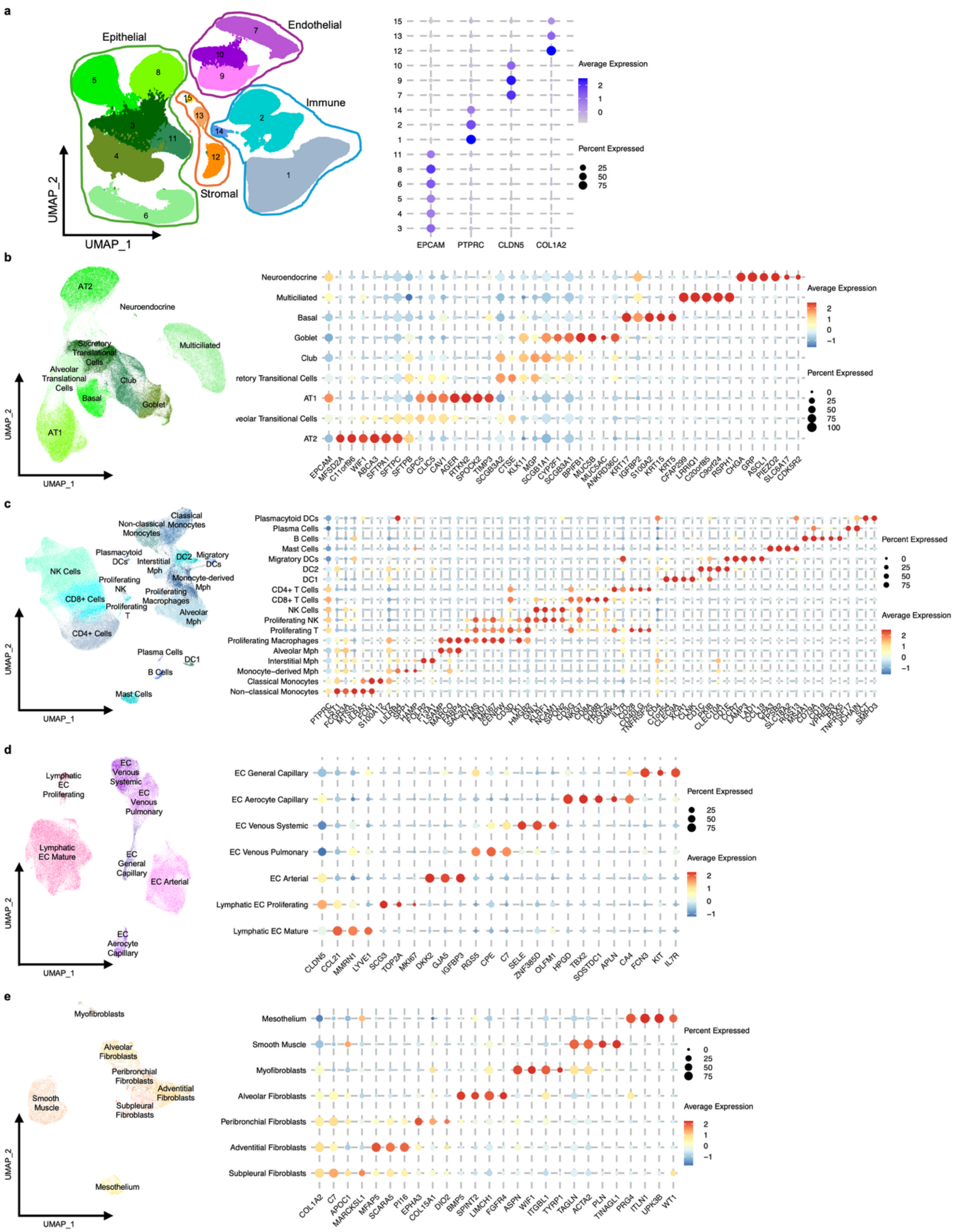

Supplemental Figure 2. Iterative clustering and cell type annotation in four lung cell categories.

bioRxiv preprint doi: https://doi.org/10.64898/2026.03.26.714500; this version posted March 27, 2026. The copyright holder for this preprint (which was not certified by peer review) is the author/funder. This article is a US Government work. It is not subject to copyright under 17 USC 105 and is also made available for use under a CC0 license.

a, Initial UMAP representation of the whole dataset. Expression of the four canonical markers for epithelial (*EPCAM*), immune (*PTPRC*), endothelial (*CLDN5*), and stromal (*COL1A2*) groups delineate the four cell categories. **b-e**, UMAP plots depicting re-clustering within cell categories: epithelial **b**, immune **c**, endothelial, **d**, stromal. **e,** Expressions of selected marker genes used for cell type annotation are shown on the right panels.

bioRxiv preprint doi: https://doi.org/10.64898/2026.03.26.714500; this version posted March 27, 2026. The copyright holder for this preprint (which was not certified by peer review) is the author/funder. This article is a US Government work. It is not subject to copyright under 17 USC 105 and is also made available for use under a CC0 license.

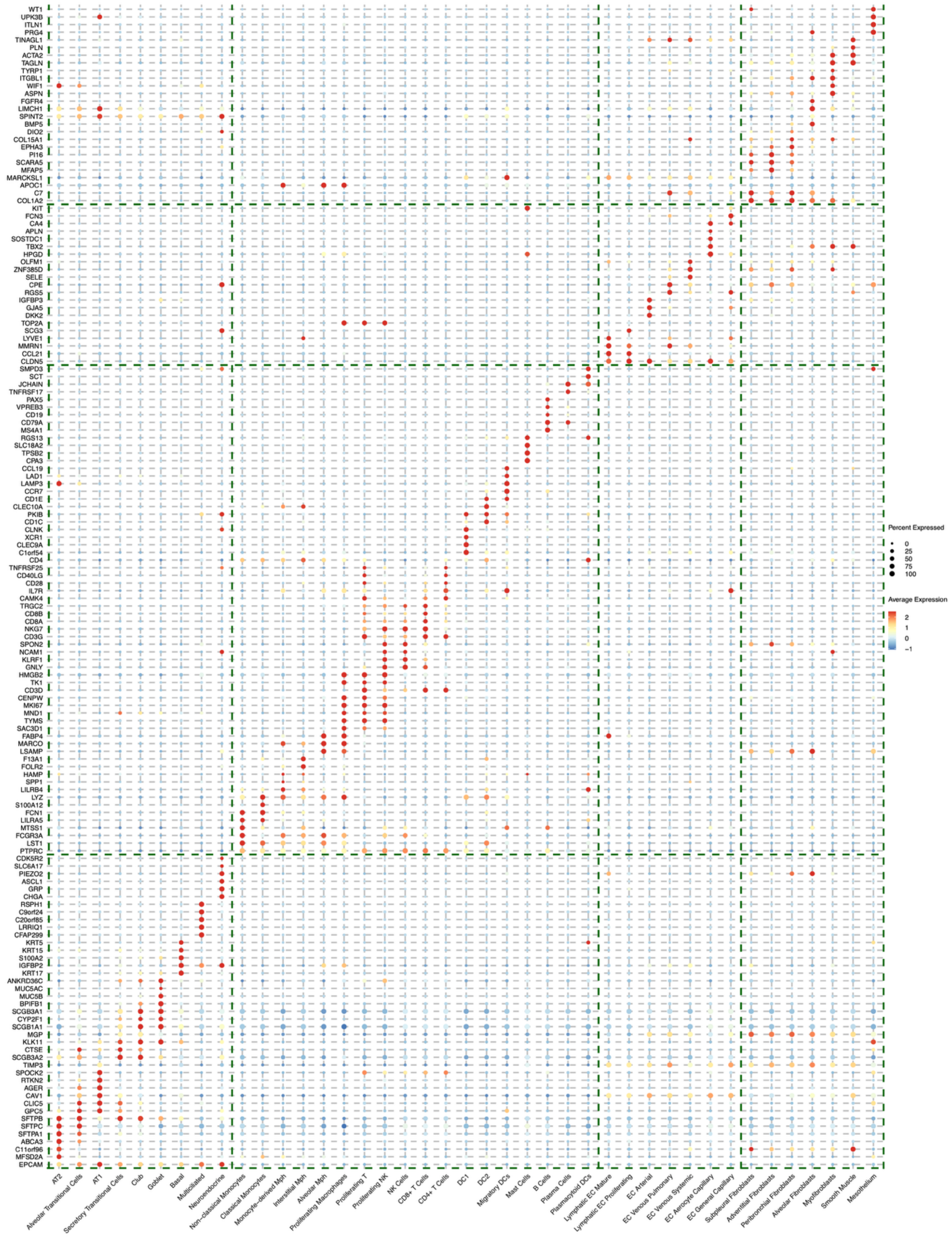

Supplemental Figure 3. Marker genes for cell type annotation

bioRxiv preprint doi: https://doi.org/10.64898/2026.03.26.714500; this version posted March 27, 2026. The copyright holder for this preprint (which was not certified by peer review) is the author/funder. This article is a US Government work. It is not subject to copyright under 17 USC 105 and is also made available for use under a CC0 license.

Dot plot showing expression of the selected final makers used to represent the 41 cell types. In this case, markers are on y-axis, while cell types on x-axis. Diagonal expression pattern shows that final set of marker genes are specific to that said cell type.

bioRxiv preprint doi: https://doi.org/10.64898/2026.03.26.714500; this version posted March 27, 2026. The copyright holder for this preprint (which was not certified by peer review) is the author/funder. This article is a US Government work. It is not subject to copyright under 17 USC 105 and is also made available for use under a CC0 license.

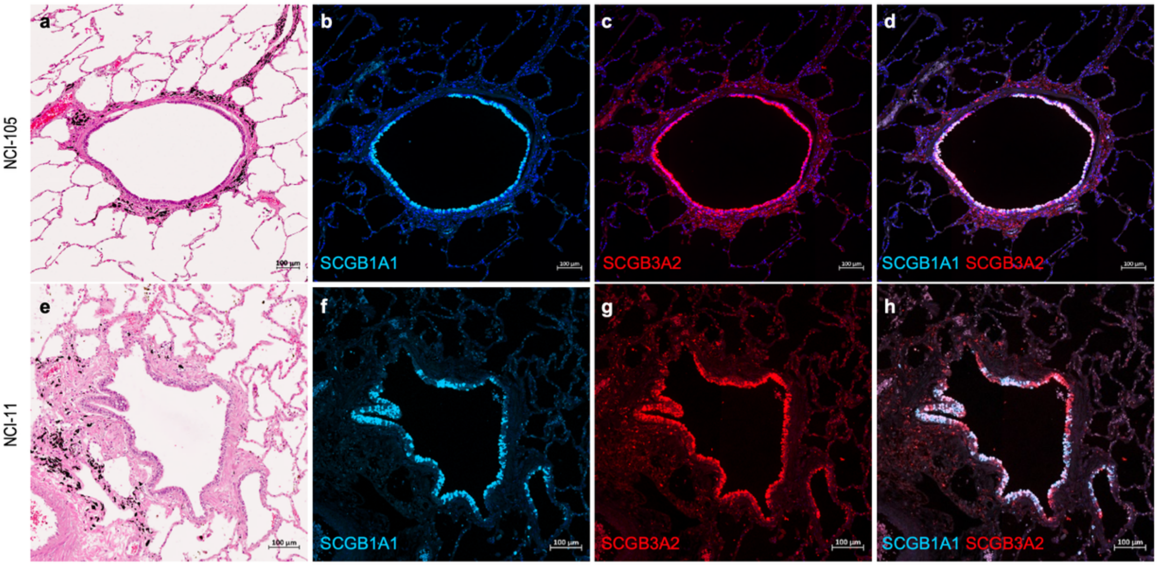

Supplemental Figure 4. Immunohistochemistry of secretory transitional cells

Shown are representative staining results for SCGB3A1 (cyan) and SCGB3A2 (red), with nuclear counterstaining (blue). **a-d** Lung tissue from a patient (NCI-105) exhibiting the lowest abundance of secretory-transitional epithelial cells, as defined by single-cell state signatures. **a,** H&E; **b,** SCGB1A1; **c,** SCGB3A2; **d,** merged. **e-h** Representative staining from a patient (NCI-11) with the highest secretory-transitional cell proportion. **e,** H&E; **f,** SCGB1A1; **g,** SCGB3A2; **h,** merged. In the merged panels, white signals along the bronchial epithelium largely reflect normal club cells. In contrast, regions that appear predominantly red represent segments with diminished SCGB1A1 signal rather than heightened SCGB3A2 levels, consistent with secretory-transitional epithelial populations. The representative patient with high secretory-transitional cell proportions displayed more extensive red-dominant segments compared with the representative low-score individual. Scale bars, 100 µm.

bioRxiv preprint doi: https://doi.org/10.64898/2026.03.26.714500; this version posted March 27, 2026. The copyright holder for this preprint (which was not certified by peer review) is the author/funder. This article is a US Government work. It is not subject to copyright under 17 USC 105 and is also made available for use under a CC0 license.

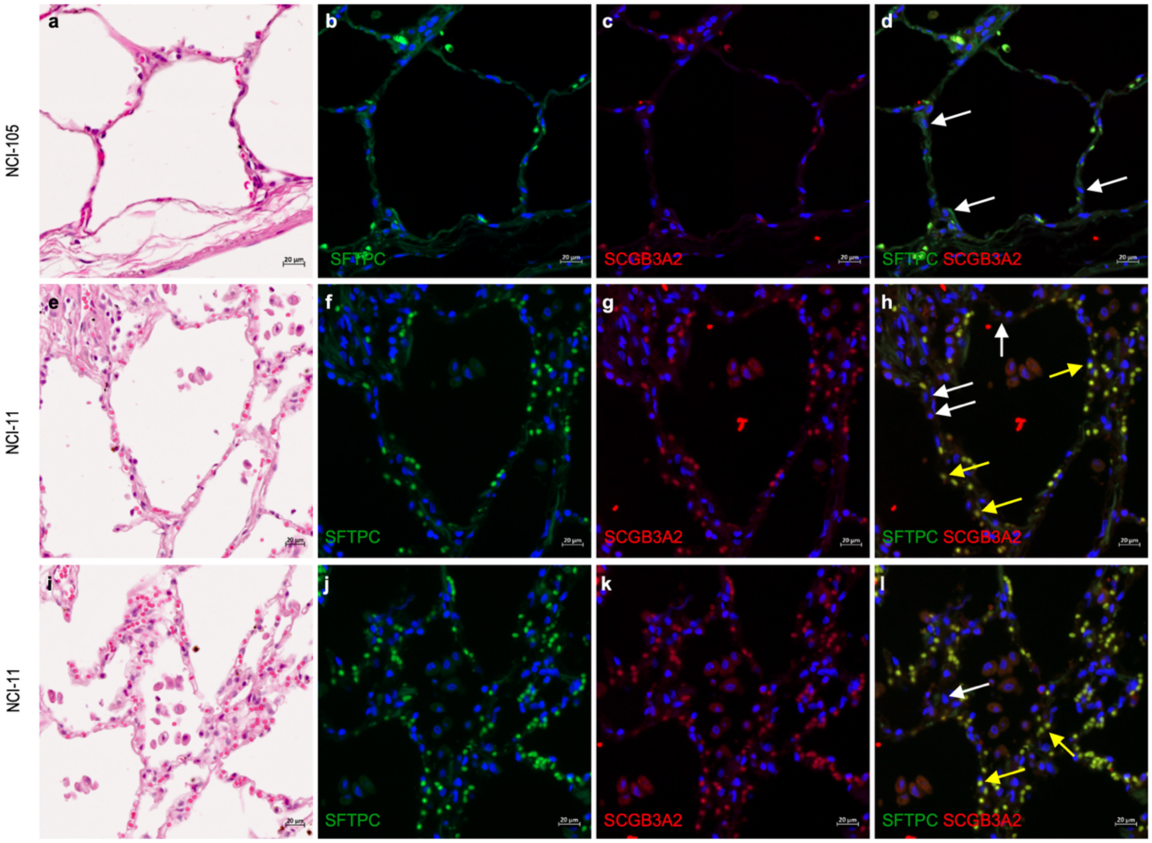

Supplemental Figure 5 Immunohistochemistry of alveolar transitional cells
Shown are representative staining results for SFTPC (green) and SCGB3A2 (red), with nuclear counterstaining (blue). **a-d** Lung tissue from a patient (NCI-105) exhibiting the lowest alveolar transitional cell proportion displays mostly normal alveolar epithelium. **e-h** and **i-l** correspond to two distinct regions sampled from a patient (NCI-11) with the highest alveolar transitional cell proportion, where transitional alveolar epithelial populations are detected. In the merged panels, white arrows indicate representative normal alveolar type II cells characterized by SFTPC signal without detectable SCGB3A2. In contrast, yellow arrows point to representative alveolar type II epithelial cells showing preserved SFTPC with visible SCGB3A2 signal, presumed to represent transitional alveolar epithelial populations. Scale bars, 20 µm.

bioRxiv preprint doi: https://doi.org/10.64898/2026.03.26.714500; this version posted March 27, 2026. The copyright holder for this preprint (which was not certified by peer review) is the author/funder. This article is a US Government work. It is not subject to copyright under 17 USC 105 and is also made available for use under a CC0 license.

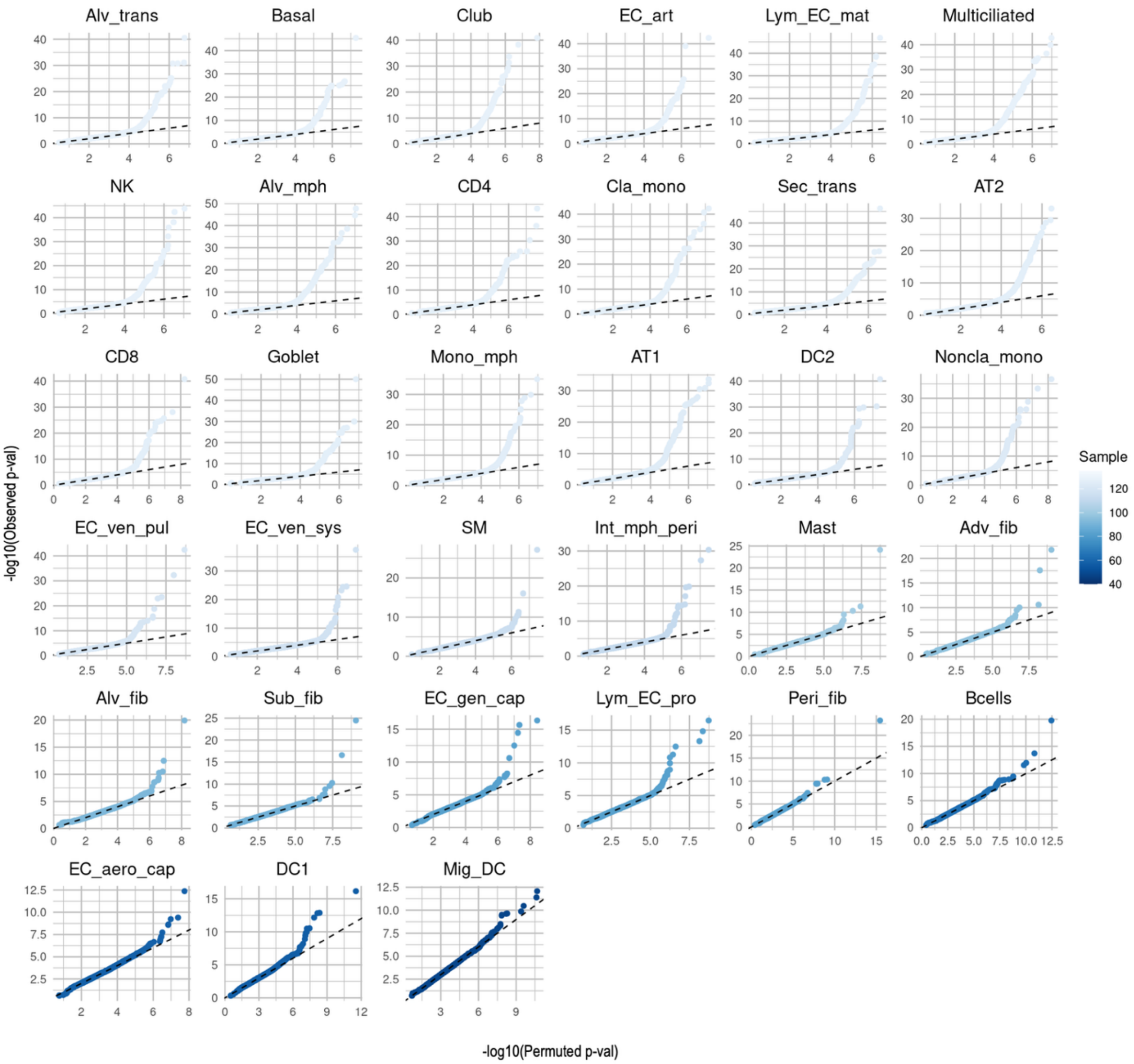

Supplemental Figure 6. Type I error control of eQTL detection

Quantile-quantile plots for each cell-type showing the observed eQTL p-values for top hit per gene (y-axis) against the permutation-based (phenotypes were randomly shuffled for each cell-type) p-values for the top hit per gene (x-axis). Shown on -$\log_{10}$ scale. Scale showing the number of individuals with >= 5 cells in each cell type.

bioRxiv preprint doi: https://doi.org/10.64898/2026.03.26.714500; this version posted March 27, 2026. The copyright holder for this preprint (which was not certified by peer review) is the author/funder. This article is a US Government work. It is not subject to copyright under 17 USC 105 and is also made available for use under a CC0 license.

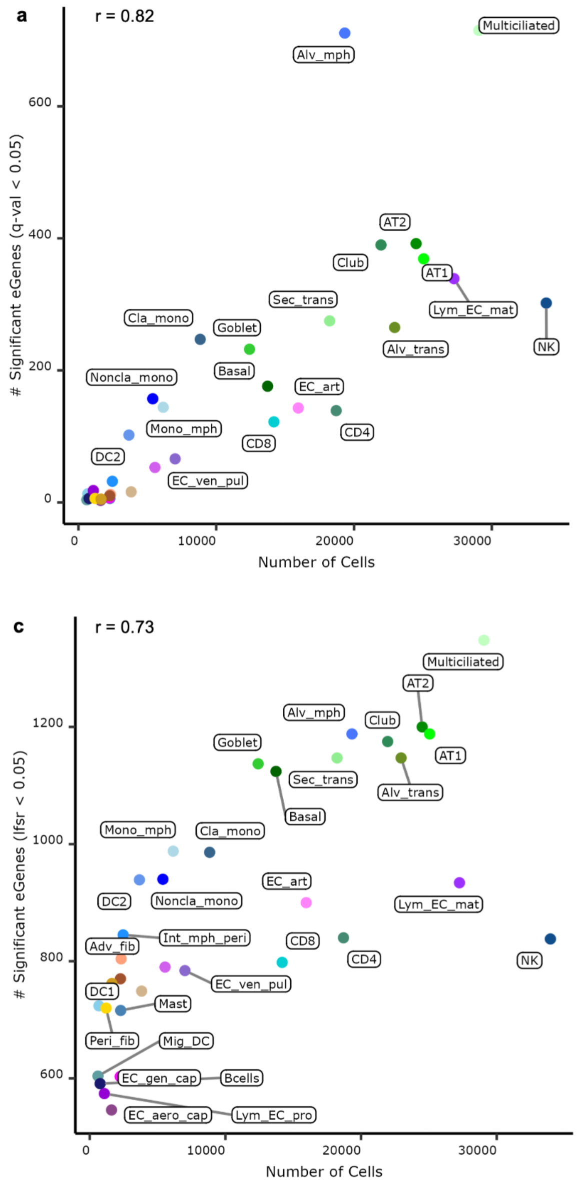

Supplemental Figure 7. eGene detection power and number of cells or individuals in a cell type

Correlation of detected eGenes (q-value < 0.05) with numbers of cells, **a,** and number of individuals, **b,** across the cell types. **c,** Correlation of detected eGenes (q-value < 0.05) with numbers of cells after eQTL effect size harmonization using mashr. Effect size harmonization improved detection power, especially for smaller-size cell types.

bioRxiv preprint doi: https://doi.org/10.64898/2026.03.26.714500; this version posted March 27, 2026. The copyright holder for this preprint (which was not certified by peer review) is the author/funder. This article is a US Government work. It is not subject to copyright under 17 USC 105 and is also made available for use under a CC0 license.

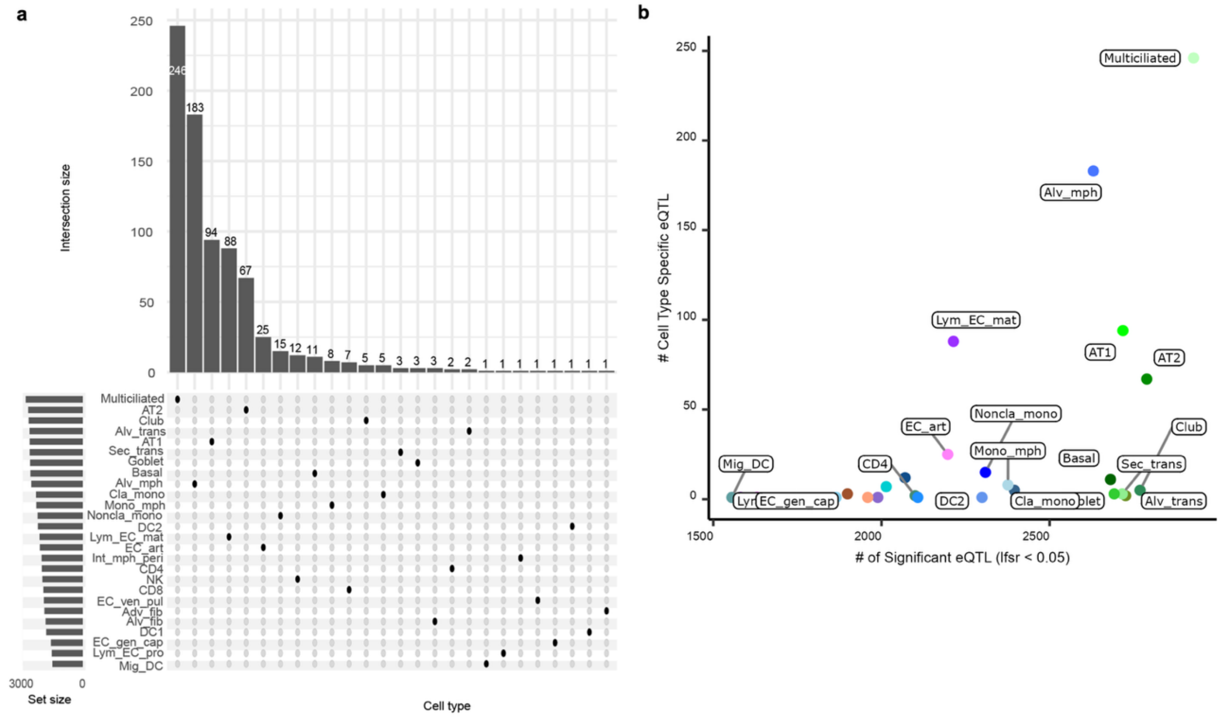

Supplemental Figure 8. Cell-type-specificity of eQTLs

a, Upset plot showing the number of cell type specific eQTLs after mashr harmonization. **b,** Scatterplot showing the number of significant eQTLs (lfsr < 0.05) vs number of cell type specific eQTLs on y-axis.

bioRxiv preprint doi: https://doi.org/10.64898/2026.03.26.714500; this version posted March 27, 2026. The copyright holder for this preprint (which was not certified by peer review) is the author/funder. This article is a US Government work. It is not subject to copyright under 17 USC 105 and is also made available for use under a CC0 license.

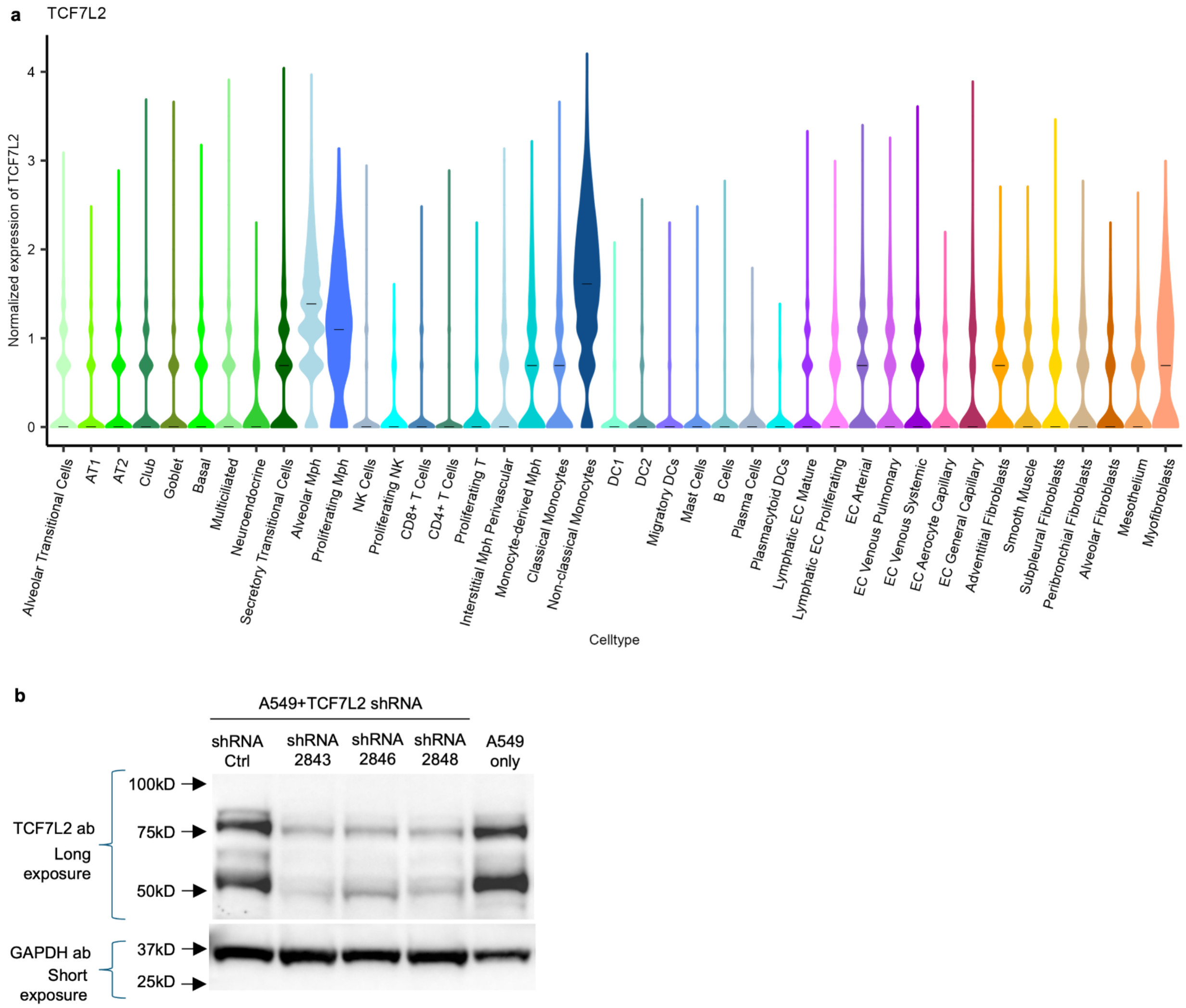

Supplemental Figure 9. *TCF7L2* levels in cell types and cell line

a, Normalized expression of *TCF7L2* across cell types are shown as violin plots. The center line denotes the median, while the violin width reflects the density. **b,** Western blotting showing levels of TCF7L2 in A549 cell lines with control or TCF7L2-targeting shRNA. GAPDH is used as a loading control.

bioRxiv preprint doi: https://doi.org/10.64898/2026.03.26.714500; this version posted March 27, 2026. The copyright holder for this preprint (which was not certified by peer review) is the author/funder. This article is a US Government work. It is not subject to copyright under 17 USC 105 and is also made available for use under a CC0 license.

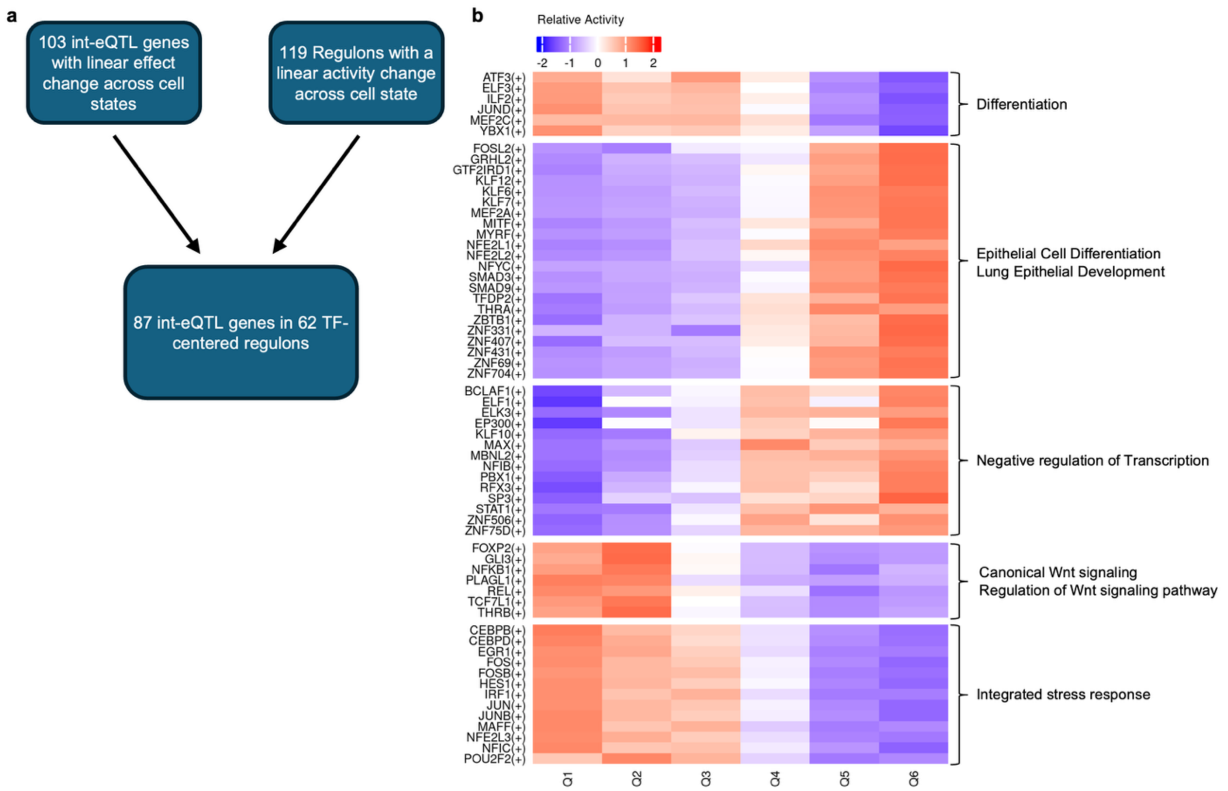

Supplemental Figure 10. Regulon activity of int-eQTL genes with a linear allelic effect change across cell states

a, Schematic of overlapping linear int-eQTL genes with linear regulons. **b,** Heatmap of relative activity of the 62 regulons which overlapped the 87 int-eQTL genes, where both regulon activity and int-eQTL effect size fit a linear model across 6 cell states. Genes were clustered into 5 clusters using fuzzy clustering using e1071 package with default settings; TF-centered regulon names shown on the left. Enriched pathways among the TFs and their associated int-eQTLs genes in each cluster are summarized on the right, highlighting overall themes of the top pathways.

bioRxiv preprint doi: https://doi.org/10.64898/2026.03.26.714500; this version posted March 27, 2026. The copyright holder for this preprint (which was not certified by peer review) is the author/funder. This article is a US Government work. It is not subject to copyright under 17 USC 105 and is also made available for use under a CC0 license.

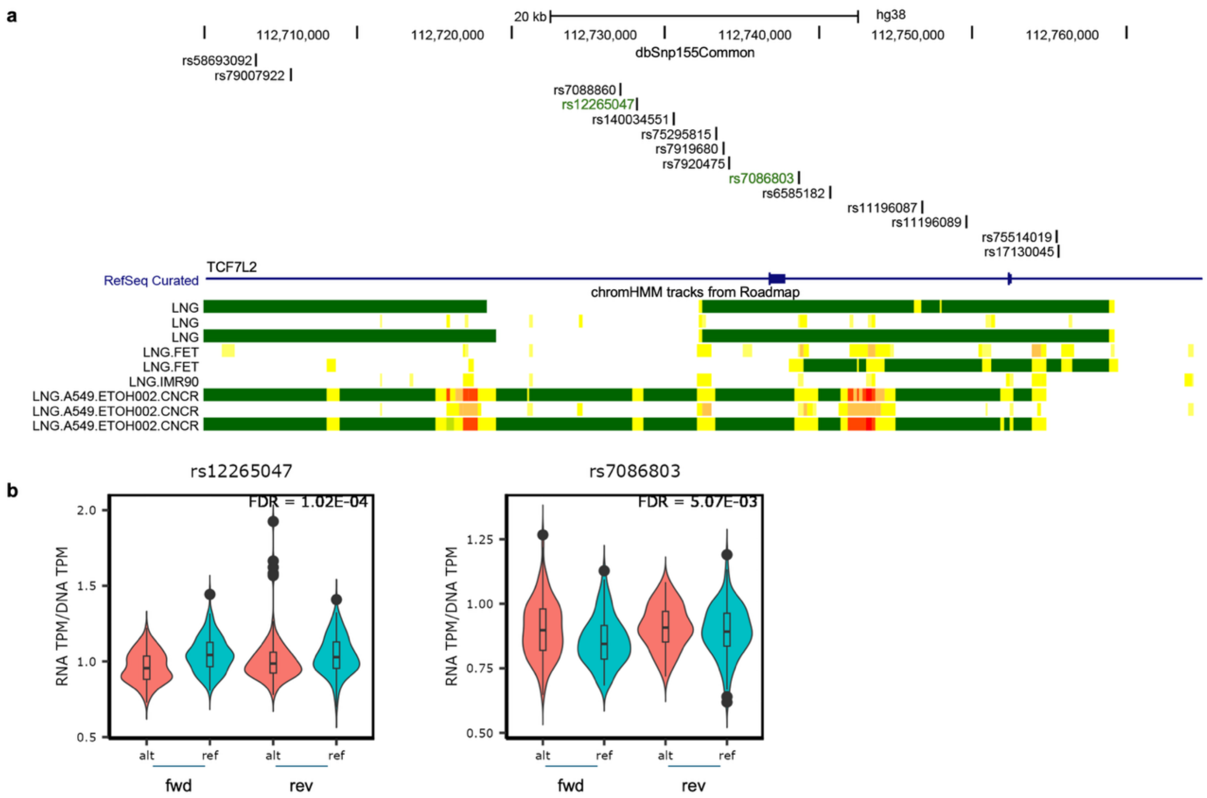

Supplemental Figure 11. Functional variant(s) nomination for *TCF7L2*

a, UCSC genome browser image showing 14 candidate causal variants (CCVs) alongside functional annotation from lung-relevant tissues and cell lines. SNPs highlighted in green are the prioritized SNPs based on the overlap with a lung enhancer (yellow shades) or promoter (red shades) and significant MPRA allelic effect in lung cancer cell line(s) (FDR < 0.01). All 14 CCVs show significant MPRA allelic function, and two highlighted variants among them overlap with functional elements in lung. **b,** Allelic effects of rs12265047 and rs7086803 on transcriptional activity (normalized) in H520, shown as violin plots. Center line denotes the median, while the 25th and 75th percentile is marked as the lower and upper line of the box, respectively. Whiskers extend 1.5 times from the 25th and 75th percentiles; outliers are represented as dots. The violin width reflects the density. TPM: Tag-per-million.

bioRxiv preprint doi: https://doi.org/10.64898/2026.03.26.714500; this version posted March 27, 2026. The copyright holder for this preprint (which was not certified by peer review) is the author/funder. This article is a US Government work. It is not subject to copyright under 17 USC 105 and is also made available for use under a CC0 license.

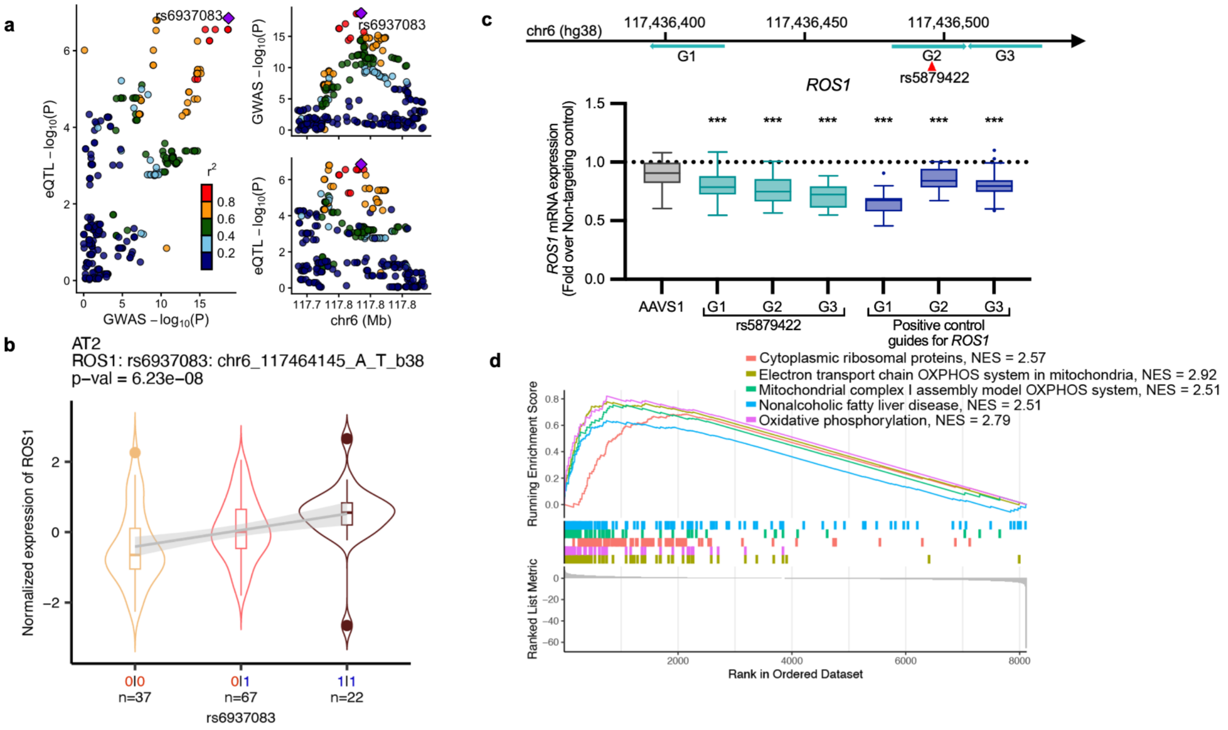

Supplemental Figure 12. Variant-to-gene validation of *ROS1*

a, Locus zoom plots of eQTL in AT2 cells for *ROS1* and GWAS (Shi et al EAS, LUAD) p-values of the region (+/- 100 kb) around the GWAS top SNP, rs68937083 in the 6q22.1 locus. LD (r2) relationships between the variants are extracted from 1000G EAS Phase 3 v5. **b,** Association between normalized expression of *ROS1* and rs68937083 is shown as violin plots. 0 = reference allele, while 1 = alternative; red indicates risk allele, while blue indicates protective. Center line denotes the median, while the 25th and 75th percentile is marked as the lower and upper line of the box, respectively. Whiskers extend 1.5 times from the 25th and 75th percentiles; outliers are represented as dots. The violin width reflects the density. **c,** Location of guide RNAs targeting the regions around our SNP of interest. Tukey plot shows *GAPDH*-normalized mRNA levels of *ROS1* in H1975 cell line from 6 replicates from three independent experiments (n =18). Fold change of target gene expression over non-targeting control is shown as median with IQR in a box. Whiskers extend 1.5 times IQR, with outliers shown as dots. AAVS1 represents a safe harbor site-targeting gRNA. P-values were calculated using a two-tailed Mann Whitney U test. **d,** GSEA analysis in AT2 cells comparing individuals that lowly (25th percentile) vs highly (75th percentile) expressed *ROS1*. The top 5 most enriched gene sets by NES are shown. NES is normalized enrichment score, where a positive score indicates enrichment of the gene set compared to the reference. *** denotes p-value < 1e-04 while ** p-value < 1e-03.

bioRxiv preprint doi: https://doi.org/10.64898/2026.03.26.714500; this version posted March 27, 2026. The copyright holder for this preprint (which was not certified by peer review) is the author/funder. This article is a US Government work. It is not subject to copyright under 17 USC 105 and is also made available for use under a CC0 license.

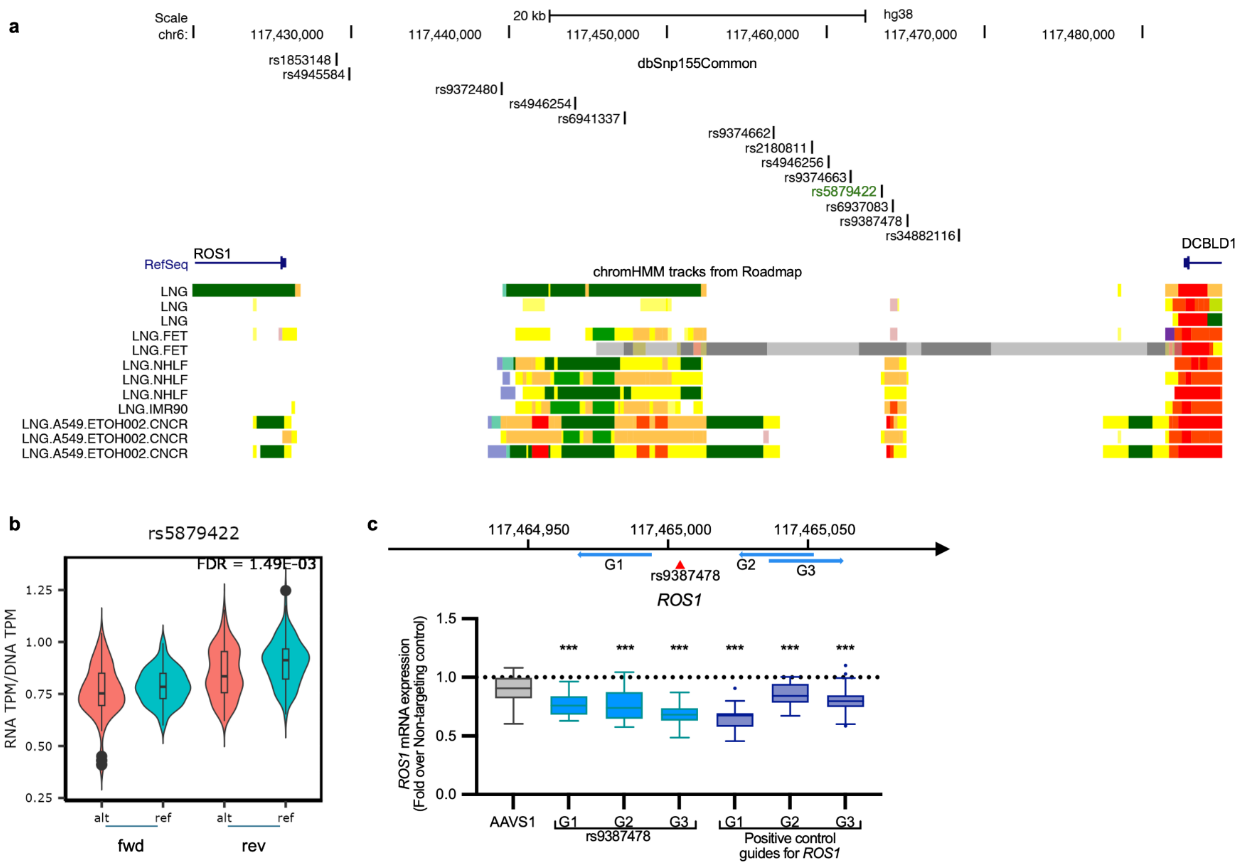

Supplemental Figure 13. Functional variant(s) nomination for *ROS1*

a, UCSC genome browser image showing 13 candidate causal variants (CCVs) alongside functional annotation from lung-relevant tissues and cell lines. SNPs highlighted in green are the prioritized SNPs based on the overlap with a lung enhancer (yellow shades) or promoter (red shades) and significant MPRA allelic effect in lung cancer cell line(s) (FDR < 0.01). **b,** Allelic effect of rs5879422 on transcriptional activity (normalized) in A549, shown as violin plots. Center line denotes the median, while the 25th and 75th percentile is marked as the lower and upper line of the box, respectively. Whiskers extend 1.5 times from the 25th and 75th percentiles; outliers are represented as dots. The violin width reflects the density. TPM: Tag-per-million. **c,** Location of guide RNAs targeting the regions around rs9387478, a CCV without MPRA allelic effect but located in the same enhancer element as the prioritized CCV, rs5879422. Tukey plot shows *GAPDH*-normalized mRNA levels of *ROS1* from 6 replicates from three independent experiments (n =18). Median with interquartile range (IQR) is shown; whiskers extend 1.5 times IQR, and outliers are shown as dots. Each dot represents the fold change of *ROS1* over non-targeting control. AAVS1 and positive control guides are replotted from Fig 6c. *** denotes p-vaue < 1e-03.

bioRxiv preprint doi: https://doi.org/10.64898/2026.03.26.714500; this version posted March 27, 2026. The copyright holder for this preprint (which was not certified by peer review) is the author/funder. This article is a US Government work. It is not subject to copyright under 17 USC 105 and is also made available for use under a CC0 license.

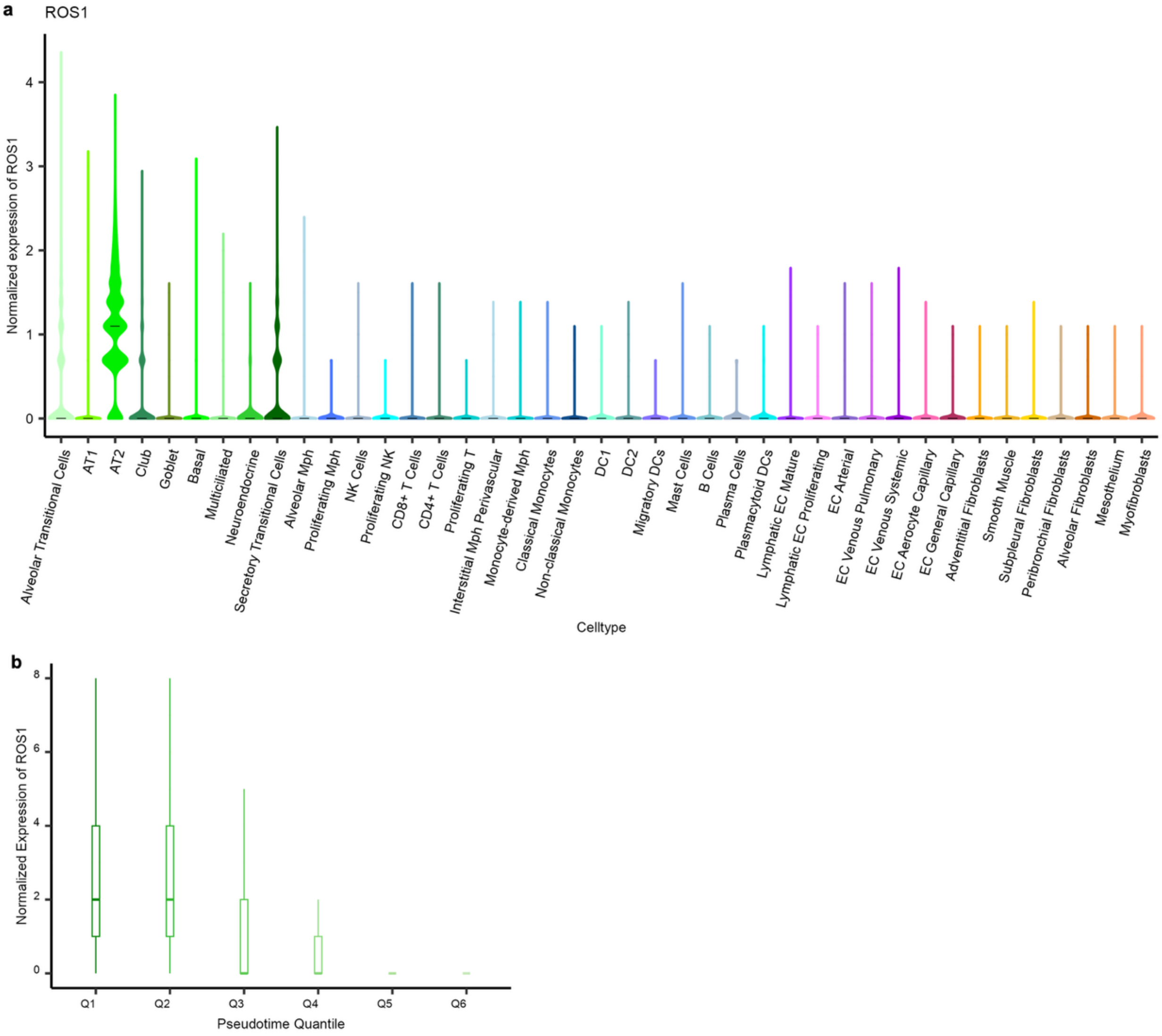

Supplemental Figure 14. *ROS1* expression is higher in alveolar progenitor/transitional cell states

Normalized expression of *ROS1* across cell types shown as violin plots, **a**, and alveolar epithelial cell states as box plots, **b**, Center line denotes the median, violin width reflects the density. Boxplots show median and the 25th and 75th percentile is marked as the lower and upper line of the box, respectively. Whiskers extend 1.5 times from the 25th and 75th percentiles. Outliers not shown.

bioRxiv preprint doi: https://doi.org/10.64898/2026.03.26.714500; this version posted March 27, 2026. The copyright holder for this preprint (which was not certified by peer review) is the author/funder. This article is a US Government work. It is not subject to copyright under 17 USC 105 and is also made available for use under a CC0 license.

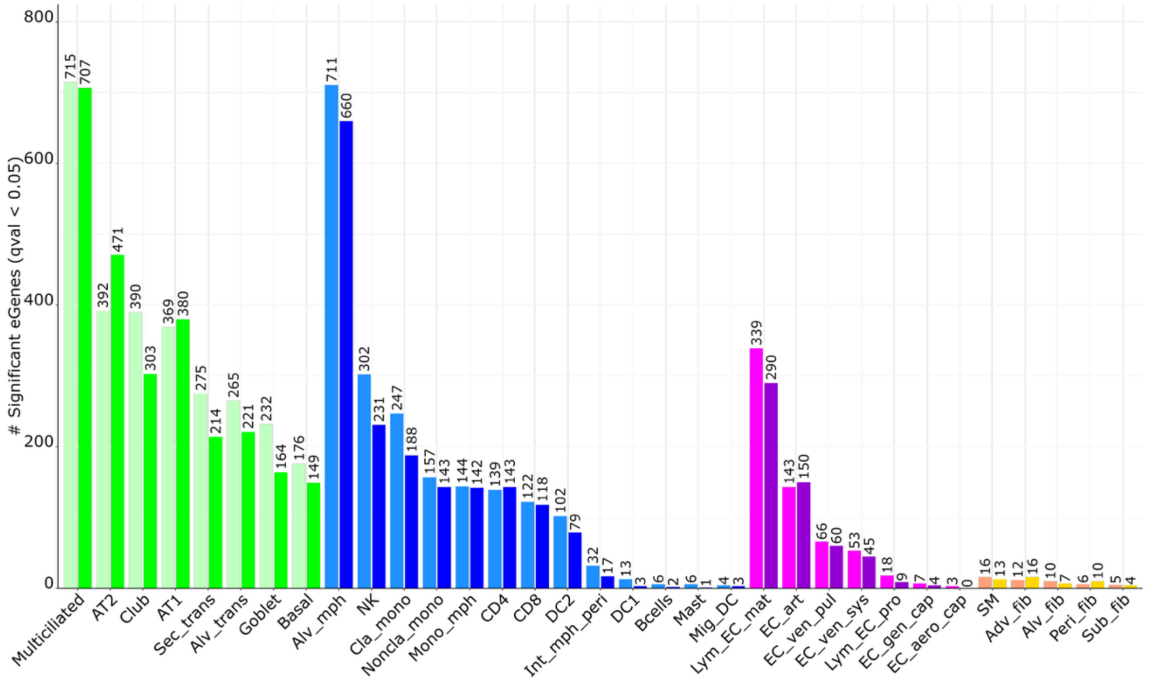

Supplemental Figure 15. eQTL detection comparisons between linear and negative binomial models

Number of declared eGenes (q-value < 0.05) across our 33 tested cell types comparing TensorQTL using linear model (left bars, lighter shades) to jaxQTL using negative binomial model (right bars, darker shades).

bioRxiv preprint doi: https://doi.org/10.64898/2026.03.26.714500; this version posted March 27, 2026. The copyright holder for this preprint (which was not certified by peer review) is the author/funder. This article is a US Government work. It is not subject to copyright under 17 USC 105 and is also made available for use under a CC0 license.

Titles of Supplementary Tables

Baseline characteristics of tumor-distant normal lung tissues collected in this study
Overview of scRNA-seq QC data after Cell Ranger analysis
Number of cells after filtering likely droplets, doublets, and low-quality
Cell type annotation based on canonical markers
Overview of number of cell types and cells per individual
Overview of cell types used for eQTL mapping
Summary of eGenes and significant eQTLs
Summary of colocalization results using EAS and multi-ancestry summary statistics
Summary of TWAS results using EAS summary statistics
Summary of lung cancer susceptibility genes identified in this study
Overview of significant dynamic eQTLs results
Beta approximation from xCELLigenece
Number of zero counts after filtering lowly expressed genes
Details of gRNA plasmids used for CRISPRi experiments
Details of shRNA plasmids used for cell growth assays
